# Regulation of germinal center B‐cell differentiation

**DOI:** 10.1111/imr.12396

**Published:** 2016-02-10

**Authors:** Yang Zhang, Laura Garcia‐Ibanez, Kai‐Michael Toellner

**Affiliations:** ^1^ Institute for Immunology and ImmunotherapyUniversity of Birmingham Medical SchoolBirminghamUK

**Keywords:** germinal center, affinity maturation, immune complex, B‐cell selection, Tfh cells, cytokines

## Abstract

Germinal centers (GC) are the main sites where antigen‐activated B‐cell clones expand and undergo immunoglobulin gene hypermutation and selection. Iterations of this process will lead to affinity maturation, replicating Darwinian evolution on the cellular level. GC B‐cell selection can lead to four different outcomes: further expansion and evolution, apoptosis (non‐selection), or output from the GC with differentiation into memory B cells or plasma cells. T‐helper cells in GC have been shown to have a central role in regulating B‐cell selection by sensing the density of major histocompatibility complex (MHC):peptide antigen complexes. Antigen is provided on follicular dendritic cells in the form of immune complex. Antibody on these immune complexes regulates antigen accessibility by shielding antigen from B‐cell receptor access. Replacement of antibody on immune complexes by antibody generated from GC‐derived plasma cell output will gradually reduce the availability of antigen. This antibody feedback can lead to a situation where a slow rise in selection stringency caused by a changing environment leads to directional evolution toward higher affinity antibody.

This article is part of a series of reviews covering Immunoglobulins: from genes to therapies appearing in Volume 270 of *Immunological Reviews*.

## Introduction

B lymphocytes are special cells, being the only known mammalian cell type that enters into somatic hypermutation, a process that leads to evolution on a cellular scale 1. This is preceded by a developmental process that already involves random mutation and rearrangements of dozens of B‐cell receptor gene segments in a stochastic manner, producing a large repertoire of B cells with a vast number of possible specificities. The initial repertoire is cleared of autoreactivities in the bone marrow, leading to an extensive naive recirculating B‐cell repertoire that still contains some autoreactivities 2. This initial repertoire needs to be able to respond to any possible challenge coming from the outside world, at least by producing B‐cell activation of weakly cross‐reactive B‐cell clones. While weak interactions are sufficient for a T lymphocyte to execute its functions 3, B cells ultimately need to be producing high‐affinity antibodies. Low‐affinity interactions are sufficient to induce initial B‐cell activation 4, 5. These low‐affinity B cells may develop without reentering B‐cell follicles 6, leading to early extrafollicular plasma cell generation that produce antibodies that can mediate efficient opsonization 7, 8. Efficient neutralization of pathogens and toxins, however, requires antibodies of high affinity. The increase in B‐cell receptor affinity from various initially activated B‐cell clones is possible. It involves clonal expansion, somatic hypermutation of the B‐cell receptor genes 9, 10 followed by selection of B cells that are of higher affinity than their precursors and do not cross‐react with autoantigens 2. We think that iterative steps of mutation and selection leading to higher and higher affinity seen in germinal centers (GC) represent nothing else than Darwinian evolution on a cellular level. Almost 30 years ago it started to become clear that these processes are occurring in GC 11, 12, 13, 14. This review summarizes processes during B‐cell activation leading to affinity maturation in the GC, with specific attention to the role of antibody during this process.

## Onset of the antibody response and development of early extrafollicular antibody

B cells activated by antigen contact undergo a complex pattern of migration directed by chemokines and chemokine‐like mediators that is leading them through several compartments of secondary lymphoid tissues (reviewed in 6, 15, 16). Antigen has to be in accessible form. Although low‐molecular‐weight antigens have access to follicles, free antigen in the absence of any adjuvant is not a good inducer of B‐cell responses 17, 18. Antibody plays an important role in uptake and distribution of antigens. Immune complexes arriving in spleens via the marginal sinus are quickly absorbed by marginal zone macrophages. B cells bind immune complexes via complement receptor 2 (CR2) and shuttle them to the center of the B‐cell follicles 19, 20. A similar process happens in lymph nodes, where antigen transported via lymph enters via macrophage‐rich areas between subcapsular sinus and B‐cell follicle, and is also transported by non‐cognate B cells 21. Free antigen is supposed to be a better inducer of memory B‐cell responses. Many memory B cells express switched B‐cell receptors. IgG not only enhances B‐cell receptor signaling but also enhances antigen presentation to T helper cells 22, 23 and therefore can lead to better plasma cell differentiation 24. However, due to the longevity of plasma cells, there is always pre‐existing antibody around at the start of a secondary antibody response. This pre‐existing antibody can immediately complex antigen, so in fact in secondary antibody responses, the antigen is never completely free of antibody or complement. Antibody and immune complex are not only important because they facilitate antigen transport into the B‐cell follicles, they will also enhance B‐cell activation of memory and naive B cells via ligation of coreceptors. Complement receptor 2 (CD21) not only plays a role in transporting antigen, it also enhances B‐cell receptor signaling via CD19 and CD81 25. The situation is more complex, as in secondary responses switched immunoglobulins (Ig) are present. IgG is less efficient than IgM to fix complement and provides negative costimulatory signals to B cells through FcγRII (CD32) 26.

Antigen‐activated naive 15, 27 as well as reactivated memory B cells 28 meet their primed cognate T‐lymphocyte counterparts at the interface between follicles and T zone, probably directed there via increased sensitivity to CCR7 ligands, CCL19, and CCL21 29. The interaction of B and T lymphocytes at the T zone–follicle border results in onset of proliferation and differentiation in the B cells. Effector cells developing from this interaction are the early extrafollicular plasmablasts – foci of plasmablasts that develop between follicles at the border between the T zone and the red pulp or in lymph node medulla 6, 7, 8, 15, 28, 30. Alternatively, B cells reenter follicles where they can form GC 16. Also during this early T–B interaction there is strong induction of immunoglobulin class switch recombination. We have demonstrated induction of Ig class switch recombination through the strong expression of immunoglobulin heavy chain transcripts once B cells interact with primed T cells at the follicle–T zone interface. This is seen at this early extrafollicular phase during primary responses 30 as well as 12–24 h after secondary B‐cell activation 28. Signals exchanged with T‐helper cells during this early extrafollicular cognate interactions include interleukin (IL)‐4 30, which in mice is the classical inducer of IgG1 class switch recombination 31. B‐cell activation including class switch recombination also happens in the absence of IL‐4 32, showing that Th2 cytokines, including IL‐13 33, are not the sole factors inducing IgG1 class switch recombination. These results were confirmed later in studies using adoptive transfers of B‐cell receptor transgenic cells and antigen‐specific T cells. By detecting actual expression of switched Ig classes early on, it was demonstrated that class switching happens very early on before GC form and before extrafollicular plasma cell foci have fully developed 34. We showed recently using TI‐II responses that extrafollicular B cell activation induces activation‐induced cytidine deaminase (AID) expression and immunoglobulin switch recombination happens during the first few days after B‐cell activation, when they have just entered the edges of the T‐cell areas 35.

Although some of the plasmablasts developing in extrafollicular foci undergo class switch recombination and express AID, there is typically no major induction of V region hypermutation or immunoglobulin affinity maturation 36. Studies by us following V region mutations by microdissection and sequencing showed very low numbers of mutations in early extrafollicular plasmablasts 37, 38. As GC are developing at the same time, we concluded that the few mutations found may be derived from early plasmablast output from close by GC. Others have detected mutations in extrafollicular plasma cells in autoimmune MRL.Fas^lpr^ mice or *Salmonella typhimurium*‐infected mice 39, 40. While autoimmune MRL.Fas^lpr^ mice produce autoreactive plasma cells, in *S. typhimurium*‐infected mice there was some affinity maturation detectable. The development of genealogical related mutated B‐cell receptor V region sequences in this area may indicate that extrafollicular plasmablast differentiation and affinity maturation are happening in extrafollicular sites. However, at least in the case of *Salmonella* infection, there are some Bcl6‐positive GC‐like structures in the basal areas of follicles 40, 41, so it is also possible that abortive GC with overactive output that never develop to normal size produce hypermutated and affinity matured output that seeds extrafollicular plasma cell foci with hypermutated cells.

Plasmablasts developing after the initial T cell–B cell interactions seem to undergo a pre‐programmed number of divisions. Experiments with different numbers of precursor cells show that plasmablasts differentiate after five to six cycles into non‐proliferating plasma cells 37. Depending on the extent of the plasma cell response, the majority of plasma cells will die by apoptosis within the next couple of days and typically a limited number of cells survive in the longer term 37. The lifespan of this limited pool of splenic plasma cells seems to be, at least in the medium term, regulated mainly by replacements coming through newly formed plasma cells, which is either new extrafollicular responses or output from GC. This leads to a slow replacement of plasma cells in extrafollicular foci over time with more and more plasma cell being derived from GC 37. Similar observations in bone marrow led to the niche hypothesis for the regulation of plasma cell survival, meaning that limited sized niches of accessory cells present in certain microenvironments do support plasma cell survival in the long term 42.

## B‐cell maturation to become a GC B cell

Some of the B cells activated during initial cognate interaction with T cells will not differentiate to form plasma cells but to reenter follicles. Re‐entry into follicles is directed by loss of CCR7 ligand sensitivity and prevailing signaling of Ebi2 43, 44. Through CXCR5 and Ebi2‐directed movements, B cells move from outer follicles toward interfollicular areas 27, 45. These are located at the edges of the T‐zone under the subcapsular sinus in lymph nodes, or in spleens at the T‐zone–red pulp bridging channels. Signals critical for GC development are exchanged in these sites 46. Loss of Ebi2 expression 44, 47 and induction of S1P2 48 then lead to B cells assembling in the follicle centers where they first form foci of proliferating blasts 49.

IL‐4 exchanged during early extrafollicular cognate interaction between B and T cells is important for the induction of GC B‐cell differentiation 50. IL‐21, produced during this phase by extrafollicular CXCR5^+^ Bcl‐6^+^ T follicular helper (Tfh) cells, seems to have a dual role supporting plasma cell differentiation on one hand, but also supporting GC differentiation and inducing Bcl‐6 expression through IL‐21R on B cells 51, 52, 53, 54. This would mean that IL‐21 acts more as a general B‐cell differentiation factor than as a factor driving differentiation in a certain direction 54.

B cells ending up in the follicle center proliferate and within days differentiate into GC displaying dark and light zones 49. It is possible that these initial follicular B blasts, similar to extrafollicular plasmablasts, undergo a pre‐programmed number of cell cycles. There are not many experiments testing GC development using different numbers of precursor cells that show an effect on GC size at an early stage of the response. Experiments were done using adoptive transfers of different numbers of 4‐hydroxy‐nitrophyl (NP)‐specific B cells from BCR knock‐in mice 55, 56. Untypical for a TI‐II antigen, NP‐Ficoll immunization of mice with artificially high numbers of antigen‐specific B cells induces strong extrafollicular plasmablast differentiation and short‐lived GC responses. GC were measured within 24 h after the onset of the follicular response and this showed a good correlation of numbers of transferred antigen‐specific B cells and GC sizes, correlating also with the size of the extrafollicular plasmablast pool 56. Other antigens however do not show this correlation. In responses to *Salmonella* there are considerable numbers of B blasts induced that migrate toward follicles and express Bcl6 40, 41. These, however, do not undergo follicular expansion leading to fully differentiated GC. It has been shown that signals through TLR4 play at least some role in this 40. Therefore, if there is a pre‐programmed set of cell divisions initiating GC differentiation, it is likely that signals such as TLR4 can abort follicular B cell proliferation and differentiation early or lead to premature differentiation of output or death.

Mature GC develop dark zones and light zones. Dark zones contain mainly proliferating cells and some tingible body macrophages 57 containing apoptotic B cells. Earliest studies already showed mitosis in the dark zones of GC 57. Ki‐67 staining on the large GC of human tonsils indicates intense proliferation in the GC dark zone 58. B cells of the GC dark zone are termed centroblasts 59. The light zone appears lighter in conventional histology because of the wider spacing between B blasts caused by the bodies of follicular dendritic cells (DC). As FDC hold antigen deposits, it was proposed that antigen‐dependent selection happens in the GC light zone 14. Because of the lower proliferative activity in the light zone, B cells here are termed centrocytes. Ki‐67 staining shows a considerable percentage of cells being in cell cycle in the light zone as well, especially in the outer light zone 58, 60 where many T helper cells are located. Some of the outer light zone T‐helper cells express preformed CD40L 60 indicating that they may have a role in centrocyte activation and selection. More recent studies using nucleotide analogue pulse‐chase labeling have clearly shown that B‐cell proliferation is triggered in the light zone, with proliferating cells rapidly moving into the dark zone 61, 62. Others have confirmed this using laser‐induced fluorescence labeling *in situ*, and also shown convincingly that this is triggered by signals from T‐helper cells in the light zone 63.

This demonstrates that migration is a major theme in GC. Light and dark zones are developing due to the balance of sensitivity to CXL13, expressed by FDC in the light zone 64, and CXCL12 expressed by stromal cells at the GC–T zone interface. Stroma in this area, extending long processes into the dark zone, produces CXCL12 65. Centrocytes that have been selected in the light zone upregulate CXCR4 expression that leads to increased migration toward CXCL12 64.

## B‐cell selection in GC

GC are sites where hypermutation specifically directed toward VDJ heavy and light chain genes is activated 12, 66. A key enzyme in this process is AID, which is already active at the earlier phase while B blasts undergo initial Ig class switch recombination. This is further upregulated once B cells express Bcl‐6 and fully adopt a GC phenotype 35. As immunoglobulin gene hypermutation should lead to random improvement or deterioration of B‐cell receptor affinity, an efficient selection process is needed to select B cells that are of at least as high affinity as their parent cells. Hypermutation is initiated repeatedly. An iterative process of mutation, proliferation, and selection is the only efficient way that may develop the complex mutational patterns that are seen at the end of the affinity maturation process 67. An iterative process is also the only way to explain the development of genealogical trees on a cellular level, with branching leading to variations of mutated Ig V region sequences that have shared mutations developing at the bottom of the tree, and branches leading off from there that share these mutations but differ in additional mutations 12, 13, 68. The development of such genealogical trees shows that reproduction through fierce proliferation, variation through Ig V region hypermutation, and natural selection for higher affinity variants drive a process on the cellular level that is the same as what happens on the level of whole organisms in Darwinian evolution.

B‐cell selection shapes the output from the GC reaction not only by shaping genealogical trees but also leading to complement‐determining regions of immunoglobulins attracting more and different mutations than the framework areas. Some of these mutational patterns are actual mutational hotspots where DNA sequence motifs are preferentially targeted by the mutational machinery. The majority is due to the effects of selection, with replacement mutations being more likely to be beneficial in the complement‐determining regions and strong selection against replacement mutations in framework areas leading to an apparent preference for silent mutations 68, 69, 70.

### Role of T‐helper cells in GC

Early theories on evolution in the GC focused on the role of antigen held on FDC for the selection of GC B cells 11, 14. Interaction between B cell receptors and antigen held here in the form of immune complexes is the obvious factor that could provide natural selection signals. The common presence of CD4‐positive T‐helper cells surrounding the area where antigen is held on FDC networks lead to the hypothesis that T‐helper cells have a key role in regulating GC selection. Early studies showed that T‐helper cells in GC express cytokines such as IL‐4 or IL‐10 that may regulate B‐cell differentiation 71, 72, 73. It was then realized that a mechanism, where antigen bound by B cells had to be presented to local T‐helper cells to avoid death by apoptosis, would be a way of how survival of newly emerging autoreactive B cells could be prevented. This led to the hypothesis that interaction of B cells with antigen held on FDC is then followed by antigen uptake and presentation to local Th cells in the outer light zone 74, 75. Indeed, it was shown that T cells infiltrating follicles are specific to the immunizing antigen 76. Further, the presence of preformed CD40L in T‐helper cells of the outer light zone shows that they are primed to interact with local B cells 60. Further evidence came from studies of T‐independent GC 56. These GC that develop vigorously for 3 days after NP‐Ficoll immunization have a very limited lifespan. Similar to GC induced by T‐dependent antigen, their B cells upregulate Bcl6, express AID 35, and lose Bcl‐2 expression making them sensitive to apoptosis. Although these GC contain plenty of antigen held on FDC that should be able to provide positive selection signals, they involute via widespread apoptosis within 3 days of forming 56. The easiest explanation for this is that at this stage the initial antigenic stimulus is not able to support survival of B cells any longer and B cells have differentiated into a stage where it is essential to receive survival signals from T‐helper cells.

Further experiments tracking mutations in these AID expressing GC, in order to obtain information about selection events, produced a disappointing result: apart from 20% of the cells carrying one or two mutations – significantly above the PCR polymerase error rate – there was not much to be seen. This was very little compared with up to six mutations found in 85% of B cells at a comparable stage of development in responses to T‐dependent antigen. This means that either, without proper CD40 stimulation from T‐helper cells the hypermutation machinery is induced only very inefficiently or, that a lack of signals from T‐helper cells in the light zone prevents recirculation into the dark zone preventing accumulation of further rounds of hypermutation 38. The frequency of mutations in these experiments in extrafollicular plasmablasts induced with TI‐II antigen is of the same low rate as in the corresponding GC, confirming that extrafollicular responses are associated with some immunoglobuline gene mutations 40. Some minor affinity maturation has been described for T‐II antigen‐induced antibody responses. It is possible that limited hypermutation in extrafollicular sites not involving GC‐like recirculation due to insufficient T‐cell help in these areas is responsible for this.

A theoretical study testing different mechanisms acting on affinity maturation using *in silico* modeling predicted that competition for T‐cell help would make the mechanism for selecting higher affinity B cells that would be most efficient in leading to rapid affinity maturation. This was more efficient than competition of individual B cells for access to antigen 77. A mechanism as to how such competition for T‐cell help might work would be that higher affinity B cells take up more antigen from FDC, or process it more efficiently and present it leading to higher concentrations of MHCII:peptide on their surfaces 78. This hypothesis has been tested recently in a study that used DEC205 antibody conjugates that are able to force entry of cognate antigens into the antigen presentation pathway of GC B cells. Combination with intravital observation and pulse labeling studies showed that cognate interaction between centrocytes and GC T cells directs B cells back into the centroblasts pool and, more importantly, that this correlates with the amount of antigen present on the surface of the B cells 63, 79. A follow‐up from this study found that the amount of antigen present during these interactions with T cells directs the extent of downstream proliferation and V‐region hypermutation 80. This proves that T‐helper cells in GC are key guardians of GC B‐cell differentiation 79. The exact nature of the signals produced by T cells that direct the return to the dark zone is still unclear. While there is a minor increase in IL‐4 and IL‐21 in GC T cells after induced cognate interactions with B cells 81, these cytokines may have a number of functions.

### The role of antibody feedback for centrocyte selection

**Figure 1:**
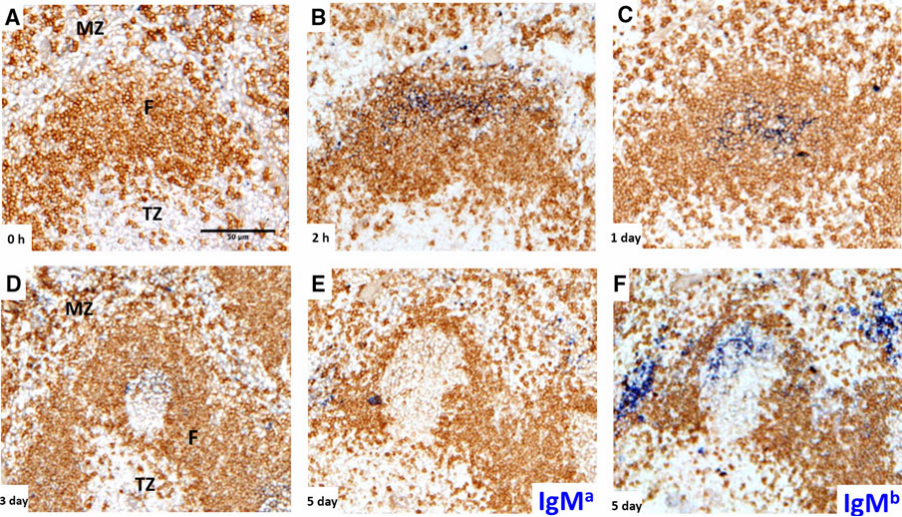
**Entry and disappearance of IgM** ^**a**^ **after immunization with anti‐** **NP** **IgM** ^**a**^ **–** **NP** **‐** **CGG** **immune complex.** Following injected IgM^a^ over the first 5 days after immunization, carrier primed IgM^b^‐expressing C57BL/6 mice were immunized with low affinity anti‐NP IgM^a^–NP‐CGG immune complex. (**A**) B‐cell follicle and surrounding area before injection showing T zone (TZ), IgD on B cell follicles (F) is stained in brown, surrounded by marginal zone (MZ). (**B**) IgM^a^‐containing immune complex (blue) is easily detectable in marginal sinus and follicle 2 h after injection, and (**C**) localizes on FDCs in the follicle center within 24 h. (**D**) Three days post‐immunization GCs have formed in the follicle center containing lacy IgM^a^‐containing immune complex on the FDC network. (**E**) Five days after immunization the originally injected low affinity anti‐NP IgM^a^ is undetectable. (**F**) An adjacent section of the same germinal center shows strong staining for endogenously produced IgM^b^ (blue, right) on the FDC networks and IgM^b^ producing extrafollicular plasma cells. Scale bar = 50 *μ*m. Further experimental detail described in 90.

While the role of T cells directing the fate of centrocytes according to the amount of antigen presented is well tested and proven, this model does not provide a satisfactory explanation why certain B cells are more efficient in taking up and presenting antigen than others. Antigen is presented in the form of immune complex on FDC predominantly, but not exclusively, in the GC light zone. It is present there in such abundance that basically every GC cell should have direct contact with copious amounts of antigen to interact with and also to take it up 82, 83 (*Figs*. 1 *and* 2).

**Figure 2:**
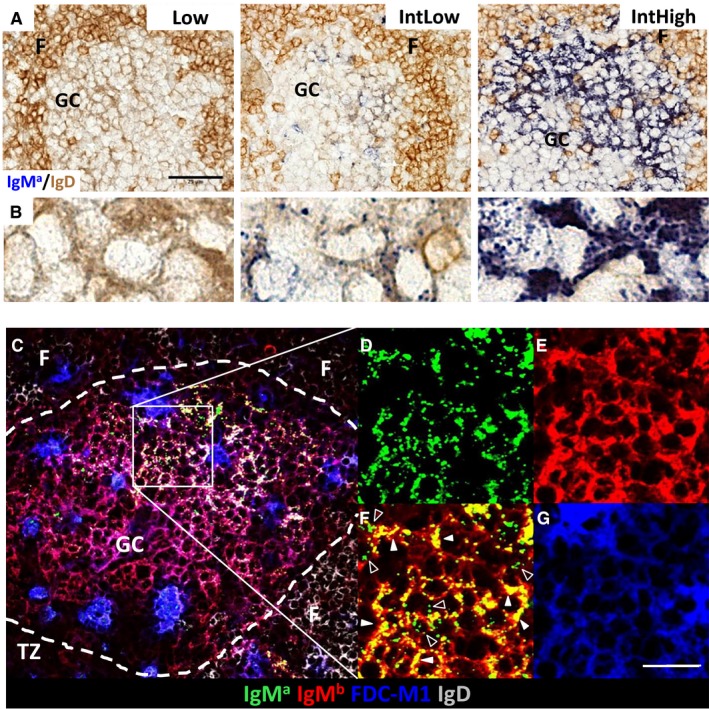
**Affinity dependence of antibody retention in immune complexes on follicular dendritic cells.** Five days post immunization using a protocol similar to *Fig*. 1, but with immune complexes containing anti‐NP IgM of Low, IntLow and IntHigh affinity. For experimental details, see 90. (**A**) Low (left), IntLow (middle) or IntHigh (right) affinity anti‐NP IgM^a^ (blue). F: follicle; GC: germinal center. Scale bar: 25 *μ*m. (**B**) High magnification detail of light zone FDC from the images above, showing punctate iccosome‐like staining remaining 5 days after injection of IntLow immune complex (middle) and iccosome plus dendritic‐like staining after injection of IntHigh immune complex (right). (**C**) Four parameter confocal microscopy 5 days after IntHigh affinity IgM^a^–IC immunization. Germinal center is outlined with dashed line. Square denotes area magnified in (**D**)–(**G**). TZ: T zone; F: follicle; GC: germinal center. (**D**) Injected IgM^a^ (green) shows mainly punctate iccosome‐like staining. (**E**) Endogenous IgM^b^ (red) is seen in a dendritic pattern on FDC and on B cells. (**F**) Double exposure of IgM^a^ and IgM^b^ shows punctate pattern (open arrowheads) mainly in green (IgM^a^ only) and some dendritic staining of immune complexes on FDC (closed arrowheads) in yellow (IgM^a^+IgM^b^) and red (IgM^b^). (**G**) FDC‐M1 staining (blue) shows dendritic FDC staining and tingible body macrophages. Scale bar: 20 *μ*m.

Development of GC coincides with the differentiation of plasma cells in extrafollicular foci that produce antibody that may complex antigen 7, 8, 30. Antigen is held in native form on FDC as immune complexes. Immune complexation is essential for transport of large antigens toward the follicle center. In lymph nodes, subcapsular sinus macrophages take up immune complexes and transport them to B cell areas and relay them to naive B cells that transport them to FDC in the follicle center 84. Similarly, in spleen, antigen is transported by marginal zone B cells shuttling between marginal zone macrophages and the follicle center 19. Both forms of transport are dependent on binding of complement component C3 in immune complexes to complement receptors first on macrophages, then on B cells, and on FDC 84, 85. Macrophages and B cells can bind C3 simultaneously through expression of different complement receptors 85. Immune complex is transferred from B cells to FDC via an active process involving actin polymerization 86. In mice as well as humans, FDC express splice variants of CR2 87, 88. Whether these have higher affinity for complement is not known.

Efficient evolution in GC can only be achieved if selection is directed toward higher affinities. Directed evolution and directional selection of species is typically caused by directional changes in the environment 89. With antigen being available in abundance, we wondered whether other factors might restrict antigen access and also lead to a directional change in accessibility of antigen. Antibody produced early on in extrafollicular foci and shielding epitopes in antigen immune complex might cause a barrier that prevents easy access by B‐cell receptors. Further, if GC would produce plasma cell output from an early stage during the GC response, and if these antibodies would transfer back and replace pre‐existing lower affinity antibodies in immune complexes inside GC, this would produce a slowly rising threshold for B cells to interact with antigen. Affinity thresholds would represent the affinities of B cells that are available in the GC at a given time, and as affinity of plasma cells would be expected to rise over time, this could provide a slowly rising threshold for antigen access that would support a slow directional evolution in GC over a long time.

To test whether such an antibody feedback mechanism plays a role, we performed experiments where immune complexes of NP‐chicken gamma globulin (CGG) and anti‐NP IgM were injected into mice 90. The antibody injected was of different allotype than the antibody produced by the mice, making it possible to differentiate between injected and endogenously produced antibody. While this experiment showed that, as seen by others, all immune complexes are quickly transported onto the FDC network in the follicle centers (*Fig*. 1), it also showed that low affinity antibody in these immune complexes was quickly replaced by endogenous antibody (*Fig*. 1). Further experiments with antibodies of different affinities showed that this replacement process is a specific effect dependent on the affinity of the specific interaction between antibody and antigen. While replacement of low affinity antibody was quite efficient, with no detectable antibody deposits left on the FDC network 5 days after immunization, high affinity antibodies were easily detectable throughout this time (*Fig*. 2). Further experiments designed to test whether such a replacement process could also happen when the GC response was ongoing showed that injected soluble IgM can deposit in ongoing GC. This was affinity dependent and also dependent on time, meaning that higher affinity antibodies were more efficient to replace endogenous antibodies and they were able to do this for longer periods, while lower affinity antibodies were only able to deposit themselves in GC early during the response. Downstream experiments to test effects of this process on selection and affinity maturation showed more apoptosis, less output, and higher affinity antibody produced due to more stringent competition from injected IgM 90.

It is not clear how IgM enters the GC and replaces pre‐existing antibody in immune complexes. Antibody binds epitopes in a reversible manner, and the speed of unbinding and rebinding to antigen determines the affinity of this interaction. Therefore, if antibody produced elsewhere is able to access the GC it should over time replace and penetrate immune complex deposits located there on FDC. GC‐derived plasma cells are located very close to the GC perimeter 90, 91, 92, and immunoglobulin secreted there may penetrate the GC simply by diffusion, but an active transport process may also be possible.

The experiments described above were all done with IgM antibody. It is not clear what the role of IgG class switching is for antibody feedback. Experiments with IgG of similar avidity showed less efficient deposition on FDC (Y. Zhang, unpublished observation), which may indicate that an active process regulates access of antibody to the GC.

Interactions of B cells with high affinity to hen egg lysozyme (HEL) with HEL coupled to phycoerythrin (PE) on FDC have been studied by intravital microscopy and *in vitro* 93. Intravital videos show these B cells capturing large clusters of material from FDC followed by B‐cell activation, and antigen capture was reduced when lower affinity interacting duck egg lysozyme coupled to PE was used. Is uptake of large clusters of antigen compatible with the antibody feedback hypothesis saying that individual affinity‐dependent interactions of B‐cell receptors determine antigen uptake and activation? Antigen can be taken off FDC in large clusters, even by ripping off some FDC membrane 93. However, B cells can only achieve this if sufficient BCR molecules are able to make contact with the immune complex. This is still dependent on these BCR molecules having higher affinity than competing antibodies. A larger number of BCR successfully binding antigen in immune complex might then either remove clusters of antigen because the BCR–antigen interaction is of higher affinity than the antigen–antibody interaction within the immune complex, or remove it from the FDC because it binds with higher affinity than the immune complex–CR2 interactions. CR2 does bind complement with modest affinity 94, meaning it should be relatively easy for B cells to remove chunks of immune complex from CR1/2 receptors on FDC. Finally, it may not even be necessary to break this interaction, as B cells may acquire parts of membrane with the antigen from donor cells 93, 95.

FDC retain captured immune complex extracellularly in a non‐processed form without phagocytosis and transfer into the lysosome antigen‐presentation pathway 83. This surface retention on FDC dendrites produces the typical lacy pattern seen in histological staining of immune complexes in GC 28 (*Fig*. 2). There is a second reservoir of membrane‐covered bead‐like structures termed iccosomes 96. Extracellular and internalized immune complex are in continuous exchange via an active process, recycling antigens from the outside toward the inside of the FDC and back 85, 86. While a substantial proportion of antigen is recycling, 60% of antigen is not recycling within 3 h 86. FDC can hold intact antigen in the form of immune complex for many months 97, 98. The presence of a second intracellular reservoir may be important, as it may represent a protected habitat where antigen is held for prolonged periods, possibly occasionally presented to reactivate B cells over sustained periods. The presence of two reservoirs is noticeable when high affinity antibody replaces lower affinity antibody during the antibody response 90. The antibody feedback experiments described above showed that after immunization with anti‐NP IgM^a^–NP‐CGG immune complex, the lacy patterned dendritic immune complex deposits were replaced by higher affinity endogenous IgM^b^ antibody quickly. While this replacement happened, a bead‐like staining pattern for the remaining IgM^a^ developed (*Fig*. 2). Immune complex in these beads, which may represent iccosomes, were far better protected from replacement by high affinity antibody over the 8‐day period the experiment was performed. If this interpretation is correct, then this would mean that iccosomes are reservoirs of longer term antigen deposition, protected from direct attack by B cells or antibody. One can speculate about the function of such a second antigen reservoir. It might be important to keep some reservoirs of antigen complexed with early‐stage antibody which is of low affinity and non‐switched, in order to stimulate or rescue B cells at later stages that are not of the highest affinity. It also has been speculated that iccosomes are sites of long‐term antigen storage providing antigen deposits for repetitive stimulation and keeping long‐term memory alive 99.

## Signals regulating output from the GC

Much is not known about signals regulating output of memory B cells or plasma cells. Plasma cells are selected for high affinity: comparing antibody affinities produced by and mutations generated in plasma cells compared to GC and memory B cells showed that the bone marrow plasma cell population showed better affinity maturation from an earlier stage of the response than the average GC or memory B cell 100, 101. This would mean that antibody feedback followed by differentiation signals from T‐helper cells may regulate plasma cell output from GC. Accordingly, we have shown that higher stringency antibody feedback, apart from leading to more cell death by apoptosis, can restrict the amount of plasma cells developing from GC 90. As stringent antibody feedback reduces the ability of B cells to take up antigen, this should lead to reduced efficiency of interactions with T‐helper cells. This was confirmed in an experiment where more stringent antibody feedback lead to a reduction in IgG1 germline transcripts – typically indicators of successful cognate interaction with T cells in the presence of Th2‐like cytokines 30. T‐helper cells in GC have been shown to produce IL‐4 and IL‐21 after cognate interactions with B cells 81. IL‐4 is known as a factor supporting Th2 responses, Ig class switching, and is important for the induction of GC 50. IL‐21 drives B‐cell differentiation and can support plasma cell differentiation as well as Bcl‐6 induction 54. As IL‐21 supports plasma cell differentiation during the extrafollicular responses 52, it is a strong candidate for doing the same for GC B cells. Studies on plasma cells generated from GC early on support a role of IL‐21 in this process (Y. Zhang, submitted).

GC used to be seen as the main producers of B cell memory. It has become clear now that memory B cells derive from cognate interaction with T cells and are not strictly dependent on signals derived from GC or Tfh cells 102. Memory B cells can be class switched or still express IgM, and class‐switched memory B cells are more prone to differentiate into plasma cells upon secondary antigen challenge 103. Markers CD80 and CD273 have been used to better characterize subsets of memory B cells and their differentiation potential 104. Earlier *in vitro* studies showed that human GC B cells rapidly adopt a memory B‐cell phenotype when cocultured with stimulated CD40L expressing memory T cells 105. Much is not known what apart from general T helper cell signals are inducing these different memory B‐cell populations 106. Research into signals driving centrocyte differentiation toward further affinity maturation, exit as plasma cells, or memory B cells certainly will be the subject of intense interest during the coming decade.

## Concluding remarks

**Figure 3:**
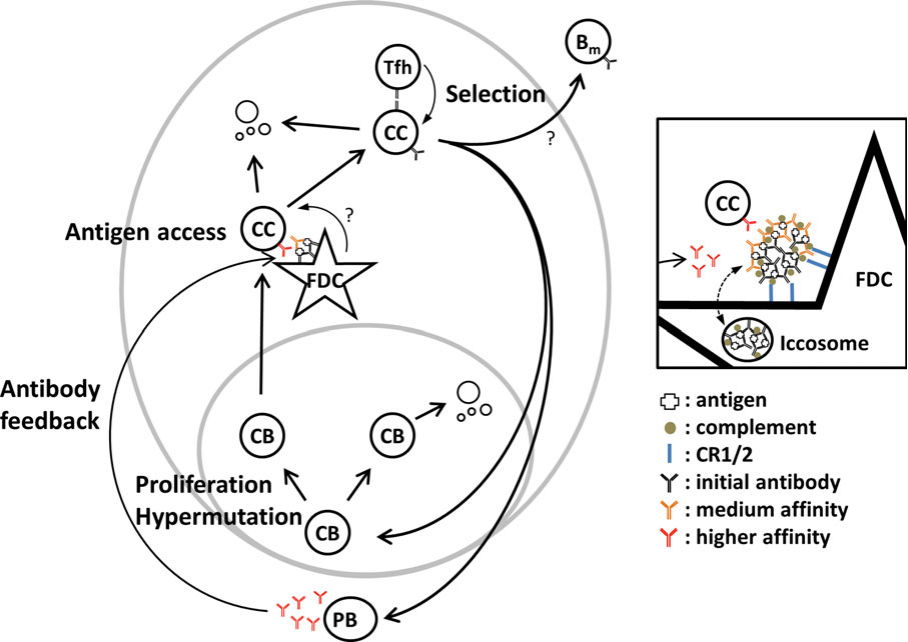
**Differentiation and selection of germinal center B cells**. Model for affinity‐based differentiation of B cells in germinal center, modified from 15, 79, including antibody feedback 90. B cells proliferate and hypermutate in the germinal center dark zone as centroblasts (CB). CB with non‐functional receptors may undergo apoptosis. CB migrate to the germinal center light zone differentiating into centrocytes (CC). These are dependent on interaction with antigen held on follicular dendritic cells (FDC). Additional signals from FDC are possible. In order to avoid apoptosis, CC must be able to take up antigen and present this to Tfh cells. Dependent on the amount of antigen presented they will receive differentiation signals from Tfh cells. Differentiation will guide them back into the dark zone CB pool, leading to further expansion and mutation, or differentiation into plasma cells leaving via the germinal center dark zone. Memory B cells may be a further result of this interaction. Germinal center‐derived plasma cells produce antibody of higher affinity that may replace antibody in immune complexes held on FDC, leading to a more stringent barrier for antigen access. Inset: FDC hold immune complex via splice variants of CR2 and Fc receptors (blue). Antigen is initially deposited on FDC complexed with low affinity antibody (black) derived from the early extrafollicular plasma cell response. Over time this is replaced by higher affinity variants (orange and red) that are produced by germinal center output cells. Some of the immune complex cycles through iccosomes, possibly representing long‐term reservoirs of antigen and antibody. Note that published experiments on antibody feedback were performed with IgM 90, not with bivalent antibody as schematically depicted here.

B‐cell differentiation in GC is regulated by a range of factors starting with antigen deposits on FDC that may or may not be accessible dependent on antibody feedback that is generated by the plasma cells derived from the ongoing GC response (*Fig*. 3). Aside from providing antigen deposits, FDC may relay further signals 107. Dependent on how much antigen B cells take up antigen from FDC and how efficiently they present this antigen, Tfh cells in the GC light zone will generate regulatory signals that will lead to further rounds of proliferation and mutation or lead to output of effector cells. Which signals exactly regulate alternative fates, and which accessory cells provide them is still unclear. Cytokines and contact‐dependent signals are obvious candidates for interventions in this differentiation process that is important not only for affinity matured responses to infection or vaccines, but is also important in the development of autoimmunity and cancer. Whether antibody feedback can contribute to tools manipulating immune responses is an open question. Our own experiments have shown that immunization with IgM immune complexes of medium affinity can lead to accelerated affinity maturation (Y. Zhang, unpublished observation). This is a delicate balance however, dependent on the exact affinity of the antibody. Finally, whether IgG and class switching have a role in regulating this process is also still unclear.
